# War and Disease. III.—The Great European War

**Published:** 1915-02-06

**Authors:** 


### WAR AND DISEASE.

### III.—The Great European War.

(Being the substance of the last of three Chadwick Trust lectures delivered by Col. F. M. Sandwith, M.D., Chairman of the British Red Cross Society, at the Royal Society of Arts on January 29.)

Colonel Sandwith said every campaign had its own peculiar medical dangers, and in a war of such gigantic dimensions as the present one these dangers were manifold, depending on the climate, the people, and even on the soil in the districts where fighting was taking place. Whilst cholera and other serious diseases were menacing the German and Russian troops, we could recognise the fact that the attention given to general hygiene, besides the value of anti-typhoid inoculation, had saved our troops from great loss from disease, and the reports which reached us concerning our men were wonderfully gratifying, considering the climatic conditions. Our sick rate at the Front was now about 3 per cent., and he believed the rate among the Indian troops was even lower. Since Sir Frederick Treves spoke at the last lecture additional figures had come in, and they were incorporated in a pamphlet issued to the soldiers by the War Office. This showed that in the first 421 cases of typhoid fever in British troops in this campaign 305 were in men who had not been inoculated. Thirty-five of those 421 cases had died of the disease, and thirty-four of the thirty-five had not been inoculated within two years, the other man in whom the disease was fatal had been inoculated only once.

### Tetanus and Preventive Methods.

But in addition we had had to suffer from special dangers which were, perhaps, not less terrible than cholera and typhoid—namely, from wounds poisoned by dirt and resulting in tetanus and gangrene. Tetanus had attacked the Allies and the opposing Germans in equal degree. It was characterised by persistent muscular tension and spasm, with periods of intensity, and of varying degree. The spasms and tension were first noticed in the neck, then in the jaw; then the face became rigid, giving the aspect of a strained grin. The muscles of the abdomen and trunk also became involved. Generally the spasms were more exhausting than painful, and the patient became bathed in perspiration. But when there was an extensive wound the spasms might produce agonising pain. The worst cases proved fatal by the third day, though a mild attack might last two or three weeks. The actual cause of death was a poisoning of the cells of the nerve centres by the bacillary toxine. The shorter the incubation period the less likely was recovery. The universality of this bacillus in the present theatre of war caused one to look back with envy on the comparatively clean soil of the South African veldt, dusty and inconvenient though that was. Tetanus was common among the ordinary population in the Aisne district.

Brilliant results had followed prophylactic anti-tetanus inoculation, this having followed on the researches of Baring and Kitisato in animals. It was clear that the earlier the dose of the serum could be given the more certain would its preventive effect be. As a rule, by the time the symptoms of tetanus were evident, the disease had obtained a serious hold on the patient; but even at this stage the treatment saved a considerable number of cases which had seemed to be doomed. It was now the practice at the Front that as soon as a wound was received the first dressing was applied, by a surgeon, a comrade, or by the soldier himself. On arriving at the field ambulance the dressing was changed, and the patient taken to the clearing hospital, where the wound was cleaned, if possible, and painted freely with a solution of iodine in spirit. Where needed, the dressing was repeated on the journey to column or base. Since November an injection of anti-tetanus serum was given at the clearing hospital or in the field ambulance to all the wounded. Thanks to the generosity of two donors—who wished to remain anonymous—a much-needed reform in the medical service of our Army at the Front had lately been added. Hitherto each man had not carried his own iodine for dressing, as the French troops did; but now every man had his supply of this in a small ampule. It was put up by English manufacturers, and was easy to carry and simple of application. The importance of this measure it was difficult to overrate.

### Gangrene and Shell Wounds.

Gangrene, which also had claimed its victims in this war, was produced in similar circumstances to tetanus; the first step was the soiling of the wound, and here the causal agent was the bacillus of malignant œdema. It was well to remember the debt we owe to the researches of Pasteur and Lister, who, once and for always, disposed of the belief that "wound fever" and "hospital gangrene" were spontaneously generated. Formerly these had been supposed to be due to stagnation of the blood at the part where the poisonous effects were seen. In France in 1868 the mortality after operations was over 60 per cent.; and a well-known surgeon at the Charité Hospital said: "When an amputation seems necessary, think ten times about it, for too often when we decide upon an operation we sign the patient's death-warrant." The bacillus of gas-gangrene, being anaërobic, did not successfully infect superficial wounds, but multiplied in deep living tissues. Shell wounds formed a good medium for it, because there was often a small wound of entry but much damaged tissue further in. But even when there was a large wound of entry, as in a case he narrated, and the wound remained largely exposed to the air, the disease supervened, though there had been instant attention, and several dressings and cleansings. After describing the usual course of a case of gas gangrene, he said these cases were very infectious, and must be strictly isolated from other wounded.

Open-air Hospitals.

His own first experience of the disease was in the Shipka Pass in September, 1877, during the Russo-Turkish War, when men were found dying of septicæmia and gangrene. There the Turkish and Greek doctors in charge had no disinfectants of any kind except some basins of quicklime on the floor. In the present war, free exposure of the wounded area to the free play of the air had produced excellent results in cases of this disease; some of our wounded were undergoing treatment in open-air hospitals. The health of our troops in all weathers showed the fine results of the open-air life. Repeated attempts had been made to supply a vaccine to counteract the bacillus of gangrene, but nothing had yet been achieved in this regard to warrant its use on man. It was hoped, however, that Sir Almroth Wright and others would be successful in their researches before long.

Anti-Frost-bite Measures.

There had been a number of cases of frost-bite among the troops, and if this condition were not attended to, gangrene supervened. As freezing was not a painful process, the unobservant man might not be aware of what was happening to him. The part must be gradually thawed by rubbing, but the process of inducing the return of blood to the tissues was a painful one. Sudden application of warmth destroyed the tissue, and paved the way for gangrene. In Napoleon's great retreat from Moscow, frost-bite was proportionately much more severe among those on horseback than among the foot soldiers. The cold during January 1878 was so intense that eighteen of Baker Pasha's sentries were frozen to death in one night, and an order was promulgated requiring the sentries to be changed every fifteen minutes. Some of the cases of frost-bite among the soldiers were grotesque in their ghastliness. Our well-fed and well-clothed soldiers were not likely to suffer so extremely from frost-bite, especially as the devoted women of England had developed a positive passion for knitting warm articles for the men. The difficulty at present was to find for the men clothing in which they could stand for hours in wet trenches at an almost icy temperature. Waders did not settle the question, because quick movement was sometimes required, whether in attack or in retreat. As to the prevention of frost-bite, he remembered asking Colonel Burnaby, who had been to Khiva, how he kept his feet warm. The reply was a laconic "I don't know," and that was all he could get out of him; but he, Dr. Sandwith, thought it likely that rubbing grease, such as did not mix with water, all over the body might be of some avail.

This struggle differed from previous wars in its uninterrupted violence. In other campaigns there had been intervals to allow troops to rest and recuperate; but the only rest in this was the result of organising a period of absence from the Front for some, while other troops took their places. Only those who had been at the Front could realise the horror and the strain of it all; with broken rest, bad weather, and the nerve-shattering effect of the huge shells. When the first batch of 230 wounded soldiers arrived at one of the London hospitals, they were so exhausted that the only thing to do was to get them to bed quickly, though some wounds were so foul that they had to be dressed immediately. All that night they slept the sleep of utter exhaustion, and they had to be roused to be washed. But after the first thirty hours they suffered from sleeplessness; they slept only very lightly, and frequently shouted out, feeling that they were still being attacked; others complained of horrible sights haunting them. They were not by any means lacking in courage, but the nerve strain had been excessive, and produced intense neurasthenia. A typical case was that of an officer who was thrown down by the explosion of a shell. Some blood escaped from his ears, and he was deaf for fifteen minutes. On reaching the nearest field hospital he collapsed, and was put to bed; he had pain at the back of his neck, and gradually developed paralysis of both lower limbs, loss of sensation to heat and cold, and there was fugitive loss of memory, difficulty in moving the tongue, and diminution of sight, especially in the left eye. The condition cleared up in six weeks, and he returned to the Front.

Pests and Disease Carriers.

The lecturer next devoted attention to the question of vermin among troops, which were not only pests in themselves, but were carriers of disease from the sick to the healthy. Bugs were bearers of certain tropical diseases, and lice were believed to disseminate typhus and relapsing fevers. The lice were found inside the men's shirts, to which they clung by some of their six legs; and the psychic disgust of cleanly men at their presence was a factor to be taken into account; some officers dreaded lice more than they feared the enemy's bullets. Instructions with the idea of minimising this danger had been issued to the Army.

With regard to typhus fever, this was at one time a common condition in poverty-stricken areas of large cities, and Ireland still had outbreaks of it. The disease was now common in Russia and the Eastern provinces of Germany; it was an acute infectious disease, highly contagious, characterised by sudden onset, a rash, delirium, and other nerve manifestations. Severe cases might be fatal in two or three days. Its mortality was from 20 per cent. to 30 per cent.

Relapsing fever was an infectious disease, caused by the presence of spirochætes in the blood. The first febrile attack usually lasted six days, after that the temperature subsided, and there followed another, and perhaps a third and fourth attack: hence the name. The mortality was not high, but the patients were incapacitated for a long time on account of weakness. It was conveyed by means of the louse, but if the patient did not scratch the lesions he did not seem to take the disease.

